# Nutrient enrichment modifies temperature-biodiversity relationships in large-scale field experiments

**DOI:** 10.1038/ncomms13960

**Published:** 2016-12-21

**Authors:** Jianjun Wang, Feiyan Pan, Janne Soininen, Jani Heino, Ji Shen

**Affiliations:** 1 State Key Laboratory of Lake Science and Environment, Nanjing Institute of Geography and Limnology, Chinese Academic of Sciences, Nanjing 210008, China; 2 Department of Geosciences and Geography, University of Helsinki, Helsinki FIN-00014, Finland; 3 Jiangsu Key Laboratory for Molecular and Medical Biotechnology, Nanjing Normal University, Nanjing 210023, China; 4 Finnish Environment Institute, Natural Environment Centre, Biodiversity, Oulu FI-90014, Finland

## Abstract

Climate effects and human impacts, that is, nutrient enrichment, simultaneously drive spatial biodiversity patterns. However, there is little consensus about their independent effects on biodiversity. Here we manipulate nutrient enrichment in aquatic microcosms in subtropical and subarctic regions (China and Norway, respectively) to show clear segregation of bacterial species along temperature gradients, and decreasing alpha and gamma diversity toward higher nutrients. The temperature dependence of species richness is greatest at extreme nutrient levels, whereas the nutrient dependence of species richness is strongest at intermediate temperatures. For species turnover rates, temperature effects are strongest at intermediate and two extreme ends of nutrient gradients in subtropical and subarctic regions, respectively. Species turnover rates caused by nutrients do not increase toward higher temperatures. These findings illustrate direct effects of temperature and nutrients on biodiversity, and indirect effects via primary productivity, thus providing insights into how nutrient enrichment could alter biodiversity under future climate scenarios.

## Abstract (web summary)

Increased temperature and nutrient pollution are key features of anthropogenic change, but their dual effects on biodiversity remain unclear. Here Wang *et al*. conduct field experiments at two mountain elevation gradients to show that temperature and nutrients have independent and interactive effects on microbial diversity.

Spatial patterns of biodiversity are a core topic in ecology; however, the mechanisms driving these patterns remain unclear. Climatic factors, especially temperature, are regarded as the main drivers underlying diversity gradients over broad spatial scales. For instance, the positive relationships between temperature and species richness prevail along gradients in elevation and latitude, which are explained by numerous hypotheses, including the metabolic theory of ecology (MTE)^1^,^2^,^3^ and productivity-diversity hypothesis^2^. In the last 100 years, the Earth has warmed by ∼0.78 °C, and global mean temperatures are projected to increase by 4.3±0.7 °C by the year 2100 (ref. 4). The changing temperatures may affect species richness because temperature covaries with primary productivity, limits the distribution ranges of species and drives speciation rates^1^,^5^. The increased temperatures may favour higher species richness, but also result in the extinction of endemic species in colder regions, such as at high elevations and latitudes^6^,^7^,^8^.

In addition, human impacts, such as nutrient enrichment, have been identified as one of the main drivers of biodiversity loss in recent decades^9^. For instance, mountainous regions are becoming increasingly impacted by settlements and transport networks^10^, and are facing more intensive forestry practices, agriculture activities, eutrophication and habitat loss. Higher temperatures and nutrient enrichment would increase the ecosystem primary productivity^11^, which could further affect species richness^12^. Thus, the interactions between climate change and human impacts on biodiversity make it difficult to predict the spatial patterns of biodiversity^13^. The typical covariance between climatic factors and human impacts^14^,^15^, such as that along elevational gradients^16^, further complicates the evaluation of their independent roles in determining biodiversity patterns^17^. The independent effects of climate change and human impact on biodiversity patterns have rarely been addressed^18^,^19^.

A promising approach to exploring climatic effects is the use of macroecological experiments (that is, large-scale field experiments) on mountainsides. This approach integrates elevational gradients with experimental manipulations of nutrient enrichment to explore the independent effects of climate and human impacts on biodiversity^20^,^21^. For instance, De Sassi *et al*.^22^ used a natural temperature gradient along elevations, combined with experimental nitrogen fertilization, to investigate the effects of elevated temperature and increased anthropogenic nitrogen deposition on the structure and phenology of a grassland herbivore assemblage. Such field experiments along natural climatic gradients can be used to disentangle climatic effects from any effects of local environmental conditions over relatively large spatial scales.

Here we conducted comparative field experiments on two mountainsides—in Norway and China—to examine the independent effects of temperature and nutrient enrichment on aquatic bacterial richness and community composition (Fig. 1a). Along nutrient and elevation (that is, temperature) gradients, we established sterile aquatic microcosms composed of lake sediments and artificial lake water, then let airborne bacteria freely colonize the sediments and water of microcosms (Fig. 1a,b). The microcosms were left in the field for 1 month before the sediments were collected, and sediment bacteria were examined using high-throughput sequencing of 16S rRNA genes. We chose bacteria as model organisms for two reasons. First, bacteria are small, abundant, diverse, essential to virtually all biogeochemical cycles, and important components of ecosystems' response to global change^23^,^24^. Second, bacteria can passively disperse over long distances and adapt quickly to changing environments due to rapid generation times and dormant-resistant stages^25^. Bacterial communities allow us to examine patterns of diversity with a high degree of experimental control and replication in natural field conditions that are subject to real species pool effects, experiments that cannot be conducted under laboratory conditions or with larger organisms within feasible time periods^26^,^27^. Moreover, our recent field survey on the study mountains indicated that nutrients were one of the main drivers of aquatic bacterial diversity^28^.

We considered three components of bacterial biodiversity: alpha, beta and gamma diversity^29^. Alpha diversity referred to the local bacterial species richness in each microcosm. Beta diversity referred to the community differentiation among microcosms. Gamma diversity referred to the species richness of each elevation (that is, temperature) or nutrient level. We quantified beta diversity with the turnover rate of the distance-decay relationship (DDR)^30^,^31^, considering the variations in community composition from one microcosm to another along temperature or nutrient gradients. We addressed the following five questions: (1) How does the temperature effect on species richness vary along a gradient in nutrient enrichment? (2) How does the nutrient-richness relationship (NRR) vary with elevation, as representative of different temperature zones? (3) How does the slope of the temperature DDR, which is the community turnover rate along the temperature gradient, vary with the gradient in nutrients? (4) How does the species turnover rate along the nutrient enrichment gradient (that is, nutrient DDR) vary with temperature? (5) How do nutrient enrichment and temperature jointly influence bacterial communities? Our results show clear segregation of bacterial species along temperature gradients, and decreasing alpha and gamma diversity toward higher nutrients. The temperature dependence of species richness is weakest at the intermediate nutrient levels, whereas the nutrient dependence of species richness is strongest at intermediate temperatures. Thus, our empirical evidence illustrates how temperature and nutrients directly affect biodiversity, and also their indirect influence via primary productivity.

## Results

### Primary productivity and pH

In our experiments, linear and quadratic models were significantly (*P*<0.05, *F*-test) fitted for most of the relationships of temperature-primary productivity, as represented by Chlorophyll *a* (Chl *a*) (Supplementary Fig. 1), which shows that primary productivity was highly correlated with temperature. Nutrient enrichment increased primary productivity more strongly at lower elevations and in the subtropical region (Supplementary Fig. 2). This finding shows that nutrient effects on primary productivity were weaker at the colder temperatures, and indicates that in a warming climate, the ecosystem productivity could be promoted more strongly than in current climate. Higher temperatures also resulted in higher water pH, especially at high nutrient concentrations (Supplementary Fig. 3). Nutrient concentrations correlated positively with water pH, particularly at low elevations (Supplementary Fig. 4). Chl *a* and pH were positively correlated at almost all nutrient levels and elevations (Supplementary Fig. 5).

### Community composition

Bacterial communities were grouped mainly by study region (*r*^2^=0.332, *P*<0.01) and elevation (*r*^2^=0.251, *P*<0.01) based on a permutational multivariate analysis of variance (PERMANOVA) (Fig. 2a). Communities were also structured by local environments. In both regions, community variations were primarily related to elevation, temperature, pH, Chl *a* and nutrients according to multiple statistical methods (that is, multiple regression analyses (Supplementary Table 1), Mantel tests, Pearson correlations (Supplementary Fig. 6) and canonical correspondence analysis (Supplementary Fig. 7)).

Interestingly, the bacterial communities at the higher elevations in China were more similar to the communities in Norway than those at lower elevations in China (lower panel of Fig. 2a, Supplementary Fig. 8), suggesting that they had more species in common. The elevational patterns of the community Sørensen similarity between each elevation in one region (that is, China) and all elevations in the other region (that is, Norway) show that the similarity significantly (P<0.05) increased and decreased toward higher elevations for China and for Norway, respectively (upper panels of Fig. 2a). These results indicate that the bacterial communities at higher elevations in China were more similar to those in Norway, and the communities at lower elevations in Norway shared more species to those in China. This segregation of species along elevations or climatic zones is, to our knowledge, the first reported for microbes, and agrees well with the classic observations of higher organisms. For instance, Linnaeus^32^,^33^ noted in his dissertation that “… on the tops and sides of such a mountain the same vegetables might grow, the same animals live, as in Lapland and the frigid zone; and in effect we find in the Pyrenean, Swiss, and Scotch mountains, upon Olympus, Lebanon, and Ida, the same plants which cover the Alps of Greenland and Lapland”. Given the long distance between the two mountains studied, our results suggest not only the high dispersal ability of bacteria, but also that ambient environments filter species at a local scale.

### Alpha and gamma diversity

The alpha and gamma diversities, that is, the species richness (that is, OTU number) of each sample (*n*=300) and experimental site (*n*=10), respectively, were 1.97 times higher in China than in Norway (*t*-test, *P*<0.001, Fig. 2b). For the Norwegian sites, both alpha and gamma diversities decreased at high elevation, whereas hump-shaped patterns were found for the Chinese sites (Fig. 2b, left panels). The different patterns imply that the effects of temperature on diversity may differ between subarctic and subtropical regions. In both regions, nutrient enrichment had consistent effects on alpha and gamma diversity, both of which decreased with increasing nutrients (Fig. 2b, right panels). This finding indicates that nutrient enrichment impoverishes microbial biodiversity, which agrees with a recent meta-analysis on richness-phosphorus relationships of macroorganisms^34^, but is in contrast to the marginal response of soil microbial diversity to nutrient enrichment at a global scale^35^. Similar to community composition, alpha diversity was correlated positively with temperature, Chl *a*, and pH in both regions (Supplementary Fig. 6, Supplementary Table 1), and these are typical drivers of microbial species richness or community composition in lakes^36^,^37^ and the ocean^2^.

### Effects of climate and nutrients on biodiversity

The results above showed that temperature, which was correlated strongly and negatively with elevation, was an important driver for both richness and community composition (Supplementary Figs 6, 7 and Supplementary Table 1). Thus, we explored how the shape of the biodiversity-temperature relationship was modified by nutrient enrichment and how the effects of nutrients depended on temperature.

We first investigated whether the effect of temperature on species richness varied along a nutrient gradient. For the 20 temperature-richness relationships (TRRs), significant (*P*<0.05) linear and quadratic models were fitted in 15 and 7 cases, respectively (Supplementary Fig. 9). This finding supports the fact that richness is strongly temperature dependent, and suggests that the elevational diversity gradients in microbes can be explained by environmental filtering or by MTE^1^. MTE provides a framework to assess how temperature affects organismal metabolisms and influences their ecology and evolution, such as rates of evolution, community composition, gradients of diversity and ecosystem processes^1^. Accordingly, log-transformed bacterial species richness is a linear function of the inverse absolute temperature (log_10_(*S*)∝*E* × (1/*kT*), where *S* is species richness, *k* is Boltzman's constant 8.62 × 10^−5^ eV K^−1^, *T* is absolute temperature in Kelvin and *E* is the slope or ‘activation energy' in eV characterizing the temperature dependence of species richness^1^. The slopes of the 15 significant linear TRRs, which represent the activation energy, *E* (Fig. 3a) and indicate the magnitude that species richness depends on temperature, varied between −0.88 and −0.18, with a mean value of −0.37±0.20. These values are similar to microbes in forest soils^38^, but are lower than the theoretical predictions of between −0.70 and −0.60 (ref. 1). The lower *E* values of bacteria compared with macroorganisms^1^ suggests that bacteria are less dependent on temperature changes, perhaps due to their high dispersal ability, rapid generation times and dormant-resistant stages^25^. The *E* values were significantly (*t*-test, *P*<0.05) more negative in Norway than in China (Fig. 3a). This finding indicates that bacteria in the subarctic region are more sensitive to temperature than those in the subtropics and may experience larger temperature-related shifts in richness under future climate scenarios.

In both regions, the temperature dependence of species richness was mediated by nutrient enrichment, shown by the fact that *E* values were closest to zero at intermediate nutrient levels (that is, ∼4.05–7.65 mg N l^−1^ total nitrogen (TN), Fig. 3a). The fact that species richness is dependent on temperature has been shown to be influenced by various factors, such as spatial scale for plants^39^. However, the mediation of nutrient enrichment on the magnitude of temperature dependence is rarely considered. Our findings clearly indicate that richness decreased faster with decreasing temperature at low or high nutrient levels than it did at intermediate nutrient levels, suggesting that the responses of bacteria to temperature changes are strongest at very low or high levels of nutrients. Therefore, at intermediate levels of nutrient enrichment, the communities or ecosystems may be most resistant to climate influence. For instance, in the eutrophic Taihu Lake in China, increased temperatures result in earlier, longer-lasting cyanobacterial blooms^40^, which further decreases aquatic biodiversity^41^. Nutrient concentrations (that is, TN) in Taihu Lake, during the years 1997–2015, were 28.6% and 7.1% higher, respectively, than the 4.05 and 7.65 mg N l^−1^ TN intermediate nutrient enrichments used in our experiment, though with a high spatial heterogeneity (Supplementary Fig. 10). A recent study in Taihu Lake showed that nutrient reductions from intermediate levels magnified the impact of extreme weather on bloom-plagued conditions^42^, which supports our finding that low nutrient levels would increase vulnerability of diversity to climate change. However, additional studies are needed to confirm whether nutrient enrichment generally affects the temperature dependence of species richness in other ecosystems (that is, terrestrial environments) and results in altered ecosystem resistance at intermediate nutrient levels, because microbial communities are also structured by their original habitat types^43^.

Second, we examined how the NRR vary among elevations representing different temperature zones. Because bacterial alpha and gamma diversity decreased at higher nutrient levels in both regions (Fig. 2b), we used the slope of the linear regression of NRR to represent the changes in species richness with nutrient enrichment. We found that species richness decreased significantly (*P*<0.05) with nutrient enrichment only at intermediate elevations (Fig. 3b,c), suggesting that species richness at intermediate elevations was more sensitive to nutrient enrichment.

Third, we used the slopes of DDR to quantify species turnover rates (that is, beta diversity) along temperature gradients and then tested how these turnover rates varied with the nutrient gradients. More negative DDR slopes indicate higher species turnover rates. In both regions, the temperature DDRs were significant (Mantel test, *P*<0.01) at all nutrient levels (Fig. 3d). In China, the species turnover rates exhibited a shallow U-shaped pattern along nutrient enrichment gradients (Fig. 3d). This pattern suggests that nutrient enrichment first slightly increases the species turnover rate until reaching intermediate nutrient levels, and the species composition becomes more spatially homogeneous at high nutrient levels. In Norway, however, the turnover rates exhibited a unimodal pattern; they responded sharply to low nutrient concentrations (that is, 0.45 mg N l^−1^) with lower turnover rates at intermediate nutrient levels (Fig. 3d). The differing response of turnover rates along temperature gradient to nutrient enrichment for the two regions highlights the potentially different community assembly mechanisms constrained by nutrient and temperature gradients. Dissimilar mechanisms of community assembly (for example, species-sorting and dispersal limitation) have also been observed for temperate and tropical forests^44^, and may also contribute to the strikingly different biodiversity gradients of these two biogeographic regions (Fig. 2b). The two patterns in turnover rate are also inconsistent with the findings of the communities in other habitats, such as the generally increasing turnover rate with increasing primary productivity observed for freshwater plankton^45^. The explanations for the inconsistency may be the potential differences in productivity gradients among studies because the nutrient gradient we considered here was extremely long. Another reason could be the different organisms studied (that is, bacteria, phyto- and zooplankton, representing contrasting trophic groups).

Fourth, we examined how species turnover rates on the nutrient gradient, quantified with nutrient DDR slopes, varied with the temperature gradient. In both regions, the nutrient DDRs of each elevation were typically significant (Mantel test, *P*<0.05) (Fig. 3e,f). The significant DDR slopes decreased significantly (*P*<0.05) toward high elevations (that is, decreasing temperature) in China, but did not decrease significantly (*P*=0.159) in Norway. This pattern indicates that the species turnover rate resulting from nutrient enrichment did not increase at higher temperatures. Our results therefore differ from the findings for other organisms, such as vascular plants^46^, which show that species turnover rates decrease toward high latitudes. This difference may have occurred because we considered temperature and nutrient enrichment as the sole primary drivers for bacterial communities, which is unlikely under natural conditions shaped by a higher number of covariant environmental drivers.

Finally, to synthesize all the findings, we conducted partial least squares path modelling (PLS-PM)^47^ to illustrate the direct and indirect effects of temperature and nutrient enrichment on richness and community composition. For richness, nutrient enrichment had negative direct effects, while temperature had positive effects (Fig. 4a,b). Temperature was the dominant factor affecting primary productivity in both regions, while nutrients and temperature indirectly affected richness through primary productivity (Fig. 4a,b). Such consistency in the underlying drivers of richness between the two regions agrees with the parallel patterns observed of the effects of temperature (Fig. 3a) and nutrient enrichment (Fig. 3b,c) on richness in Norway and China. These results suggest that both temperature-related kinetic mechanisms^1^ and productivity-diversity hypothesis^2^,^48^ may explain the variation in species richness, while the latter appears to be the stronger factor. For community composition, nutrients and temperature exerted indirect effects through primary productivity, and primary productivity was the dominant driver in the subarctic region (Fig. 4c,d). However, in the subtropics, the direct effects of temperature were dominant and nutrient effects were weakest (Fig. 4c,d). These contrasting mechanisms are in agreement with the differences in the patterns of temperature DDR slopes along nutrient gradients between the two regions (Fig. 3d).

## Discussion

To further elucidate the interactive effects of temperature and nutrient enrichment on biodiversity, future studies are encouraged to consider different taxonomic groups, various habitats, and even more advanced experimental designs. For instance, relevant comparison of communities of micro- and macroorganisms^49^, or multiple habitats^43^, such as the overlying water and sediments in our microcosms, will go a long way toward supporting broader conclusions regarding the effects of temperature and nutrients on biota. Although microbial experiments are not appropriate for all ecological questions, microbial manipulation experiments, for example, Vannette and Fukami^50^, offer a complementary approach to field and laboratory studies of macroorganisms^27^. Furthermore, multiple analytical approaches of biological analysis, such as metagenomics or Geochip^51^, would be helpful for understanding the effects of temperature and nutrients on the functional diversity and various activities of communities^35^,^52^ and, consequently, their impacts on the ecosystem functioning and services, which often depend on biodiversity^11^,^53^. The duration of our experiments was one month, which is similar to that of previous microbial manipulation field studies investigating the underlying processes of community assembly^26^. Future experiments with a high-resolution time series and longer duration (for example, the 30-year and 150-year fertilization experiments on plant^54^ and microbial biodiversity^55^, respectively) and more global distribution (for example, Nutrient Network^56^) would provide more evidence for the dynamic patterns of the effects of global change on global-scale biodiversity.

Collectively, we answered five specific questions regarding the effects of temperature and nutrient enrichment on bacterial biodiversity. By conducting experiments along climatic gradients, we have presented the first empirical evidence of the patterns and pathways of the effects of temperature and nutrient enrichment on biodiversity in subtropical and subarctic regions. For over two centuries, ecologists have documented the relationships between biodiversity and temperature^1^,^2^,^3^,^32^, productivity^2^,^12^,^45^,^48^,^57^ or anthropogenic impacts^19^,^35^,^56^,^58^,^59^. The independent and interactive effects of these factors are central to understanding the underlying mechanisms responsible for the generation and maintenance of biodiversity, and furthermore, to forecasting the effects of global changes on biodiversity^19^. We believe our findings have important implications regarding these pivotal effects on biodiversity.

First, our results highlight the fact that macroecological experiments along environmental gradients (for example, mountain elevation gradients) are an important tool in ecological research because they allow for the disentangling the effects of individual environmental drivers on biodiversity, the independent effects of which are not be easily separated due to their covariance in nature. The current findings using microbes as model organisms offer strong examples of the importance of the study of global changes using integrating experiments and natural environmental gradients, and illustrate an emerging approach which can be distributed globally to advance our predictive understanding of ecological trends and responses. Second, temperature and nutrients play pivotal roles in maintaining elevational biodiversity patterns such that the temperature dependence of species richness is strongest at very low and high nutrient enrichment, while the effect of nutrients on species richness is strongest at intermediate temperatures. We found clear segregation of bacterial species along temperature gradients (or climatic zones), and decreasing alpha and gamma diversity toward higher nutrient levels. We documented the direct effects of temperature and nutrient enrichment on biodiversity, and also showed that both factors indirectly affected communities through primary productivity. Thus, we fill the knowledge gaps in how well we understand the direct and indirect effects of climate change and human impacts on the spatial patterns of biodiversity, and provide thoughtful insights into how nutrient enrichment may alter biodiversity under future climate scenarios.

## Methods

### Experimental design

**Figure 1:**
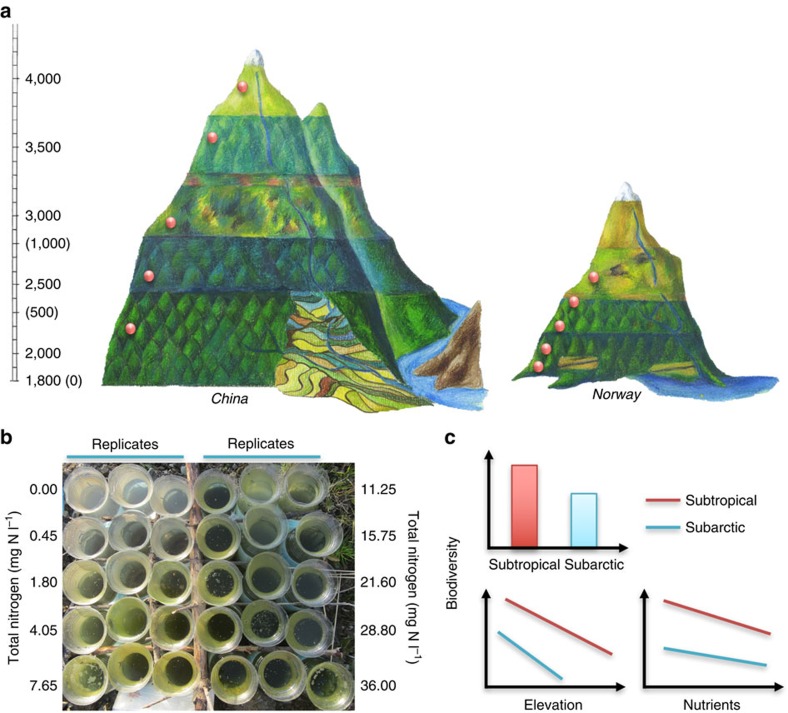
The manipulation of nutrient enrichment along elevational gradients. The experiments were conducted in parallel in the mountains of the subtropical (that is, China, left panel) and subarctic (that is, Norway, right panel) regions (**a**). The figures of the two mountains were created according to the plant species and climate zones along elevational gradients. Elevations (m a.s.l.) are shown without and with parenthesis for subtropical and subarctic regions, respectively (**a**). Along each mountainside, sterile microcosms with ten nutrient levels and three replicates at each level (**b**, field photo) were set up at each of five elevations, indicated by the brown dots (**a**), and were left in the field for 1 month. The nutrient levels were indicated by nitrogen because the ratio between nitrogen and phosphorus was consistent (**b**). Airborne microbes freely colonized the sterile habitats. Nutrient addition promoted the growth of algae, which caused gradual changes in green colour with higher nutrient enrichment (**b**). The bacterial biodiversity was expected to be higher in the subtropics than in the subarctic region (**c**, upper panel), and showed predictable patterns along elevation (that is, temperature) and nutrient enrichment (**c**, lower panels). The slopes of biodiversity along elevational gradients (**c**, left-lower panel) and nutrient enrichment (**c**, right-lower panel) were expected to vary between regions, and with nutrient levels and elevations, respectively.

**Figure 2:**
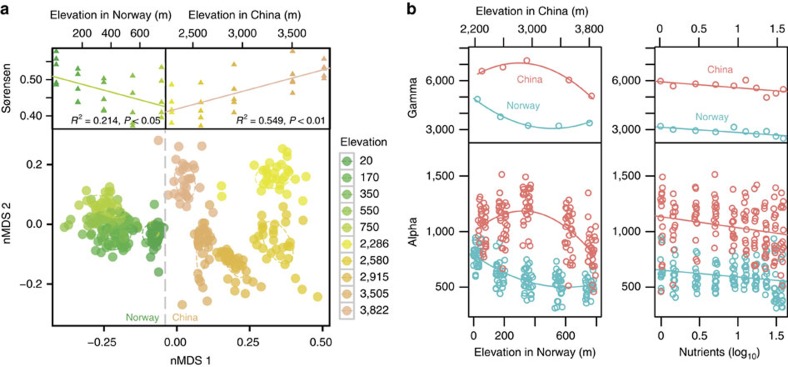
Responses of community composition and diversity to elevation and nutrients. (**a**) Non-metric multidimensional scaling (nMDS) plot of bacterial communities (lower panel), grouped by elevation (m a.s.l., indicated by colour, with higher elevations in warmer colours) and country (indicated by dotted grey line). This plot illustrates that the communities at lower elevations in Norway (or higher elevations in China) were more similar to communities in China (or Norway) than the communities at higher elevations in Norway (or lower elevations in China), which is quantitatively supported by the upper figure panels (left: Norway; right: China) that have triangle points and linear regression lines. We calculated the community Sørensen similarity along the elevational gradient between each elevation of one region (that is, China) and all elevations of the other region (that is, Norway). The relationship between the similarity and elevation was fit and tested with a linear model and permutation tests in the R package lmPerm (v.1.1-2). (**b**) Gamma diversity (upper panels) and alpha diversity (lower panels) along elevations (left panels) and nutrient enrichment levels (right panels). For diversity-elevation and diversity-nutrient relationships, we applied quadratic and linear models, respectively, and significances of the relationships were examined with F-statistics. For gamma diversity-elevation relationships in Norway and China, the adjusted *R*^2^ values were 0.952 (*P*=0.024) and 0.957 (*P*=0.022), respectively. For alpha diversity-elevation relationships in Norway and China, the adjusted *R*^2^ values were 0.518 (*P*<0.001) and 0.335 (*P*<0.001), respectively. For gamma diversity-nutrient relationships in Norway and China, the adjusted *R*^2^ values were 0.546 (*P*=0.009) and 0.332 (*P*=0.047), respectively. For alpha diversity-nutrient relationships in Norway and China, the adjusted *R*^2^ values were 0.047 (*P*=0.005) and 0.049 (*P*=0.004), respectively. The elevations (m a.s.l.) in Norway (blue) and China (red) are shown along the bottom and top axes (**b**, left panels), respectively. The amount of NO_3_^−^ (mg N l^−1^) initially added to the microcosms represents the nutrient enrichment (**b**, right panels). The points were jittered for better visualization (**b**, lower panels).

**Figure 3:**
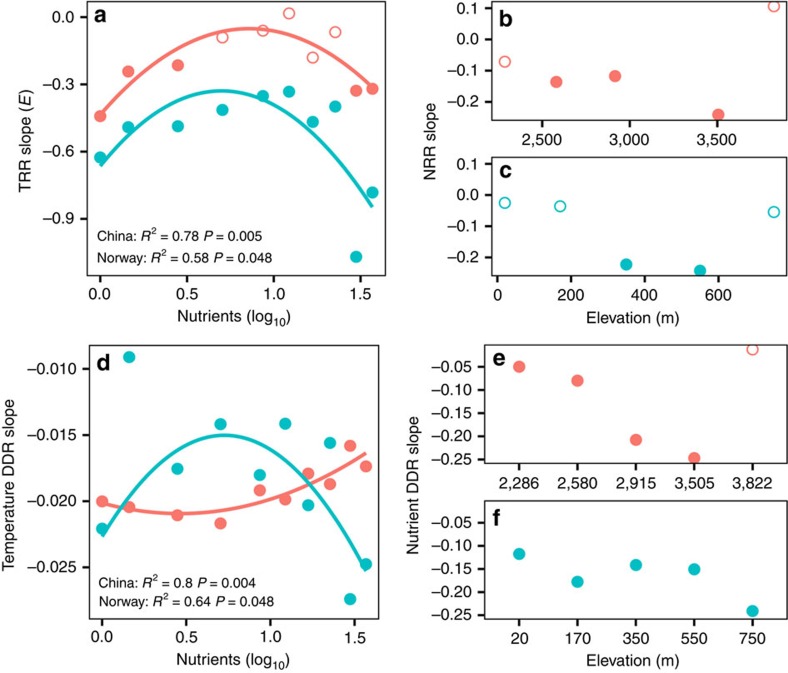
The variation of the temperature or nutrient dependence of biodiversity. Species richness plots (**a**–**c**): the slopes of the TRR and NRR along nutrient enrichment (**a**) and elevation gradients (**b**,**c**), respectively. The TRR slope (**a**), characterizing the temperature dependence of species richness, was calculated according to MTE^1^, and log-transformed bacterial species richness is a linear function of the inverse absolute temperature (log_10_(*S*)∝*E* × (1/*kT*), where S is species richness, *k* is Boltzman's constant 8.62 × 10^−5^ eV K^−1^, *T* is absolute temperature in Kelvin and *E* is the slope or ‘activation energy' (**e**) in eV. Community similarity plots (**d**–**f**): The slopes of the temperature DDR and nutrient DDR along nutrient enrichment (**d**) and elevation gradients (**e**,**f**), respectively. DDR was based on Sørensen similarity. The nutrient DDR slopes were multiplied by 100 for better visualization. Solid dots indicate the significant (*P*<0.05) relationships. Initially added NO_3_^−^ (mg N l^−1^) represents the nutrient enrichment. Blue and red dots represent Norway and China, respectively.

**Figure 4:**
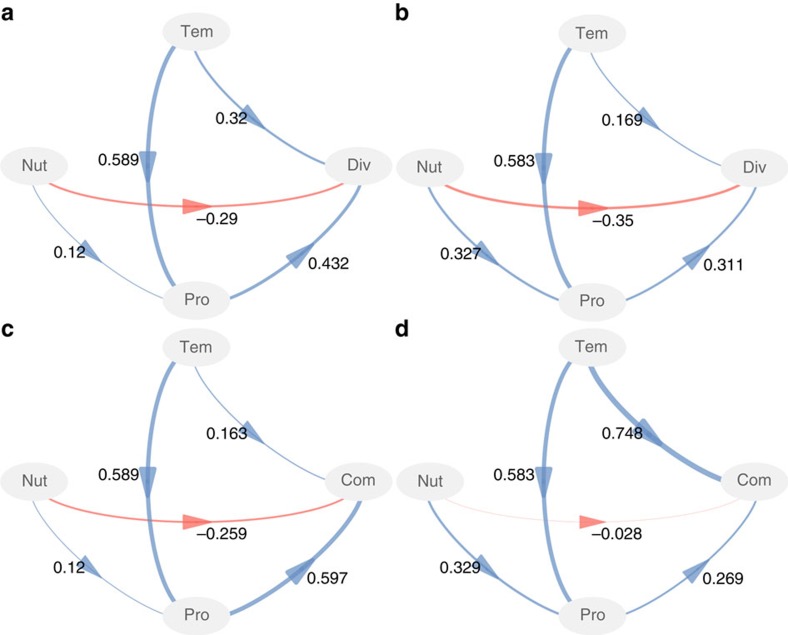
The direct and indirect effects of temperature and nutrients on biodiversity. The effects of temperature (Tem), nutrient enrichment (Nut) and primary productivity (Pro) on bacterial diversity (Div) and community composition (Com) for Norway (**a**,**c**) and China (**b**,**d**), explored with partial least squares path model. For diversity (**a**,**b**) and community composition (**c**,**d**), species richness and the first axis of nMDS were used as observed variables. For temperature, water temperature and its squared value were used. For nutrient enrichment, the observed variables included the initial levels of added NO_3_^−^ and measured NO_3_^−^, NO_2_^−^ and PO_4_^3+^. For primary productivity, the observed variables were pH and Chl *a*. Shown are the path coefficients calculated after 1,000 bootstraps. Models were assessed using goodness of fit (GoF) statistic. The GoFs for A-D are 0.630, 0.520, 0.622 and 0.718, respectively.

The parallel field experiments were conducted in a subarctic region, Balggesvarri Mountain in Norway (0–1,270 m a.s.l.), and in a subtropical region, Laojun Mountain in China (2,280–3,820 m a.s.l.)^28^, in July and September–October 2013, respectively (Fig. 1a). The climate in the Balggesvarri Mountain region is subarctic, with a growing season of ∼3 months. The annual temperatures ranged from −2.9–0.7 °C, with July temperatures ranging from 8 to 16 °C. The tree line is located at ∼550 m a.s.l. The climate in the Laojun Mountain region is subtropical. The annual temperatures ranged from 4.2–12.9 °C, with July temperatures varying from 17–25 °C. The tree line is located at ∼4,200 m a.s.l.

Along the side of each mountain, we selected unshaded locations at five different elevations. At each elevation, we set up 30 1.5 l bottles, which included ten nutrient levels and three replicates of each level (Fig. 1b). The elevations were 3,822, 3,505, 2,915, 2,580 and 2,286 m a.s.l. for China, and 750, 550, 350, 170 and 20 m a.s.l. for Norway (Fig. 1b). The bottles of different nutrient levels and replicates were arranged non-randomly at each elevation (Fig. 1b). The bottom of each bottle (∼10% of the total bottle height) was buried in the local soil. We filled each bottle with 1.2 l sterilized freshwater and 15 g sterilized sediments. The sterilized sediments were prepared before the field experiments and were collected from the centre of Taihu Lake in October 2012, freeze dried, and stored at −20 °C. The sediments were autoclaved eight times at 121 °C for 30 min, dried at 110 °C for 24 h, homogenized, and then aseptically canned with 15 g sediments per bottle for the field experiments. The dried sediments were verified to be sterile by negative DNA amplification using bacterial primers after DNA extraction following the steps in the section ‘Bacterial community analyses'. No amplification results were observed. The artificial freshwater was prepared with sterilized MilliQ water and autoclaved at 121^o^C for 30 min, and the following salts were added: CaCl_2_ 7.55 g l^−1^, MgSO_4_·7H_2_O 6.78 g l^−1^ and NHCO_3_ 3.53 g l^−1^. To facilitate the initial colonization of heterogenetic microbes, 0.91 g l^−1^ glucose was added. KNO_3_ was added at rates of 0.00, 0.45, 1.80, 4.05, 7.65, 11.25, 15.75, 21.60, 28.80 and 36.00 mg N l^−1^ to generate ten nutrient levels including the control of 0.00 mg N l^−1^. To compensate for the nitrate additions, KH_2_PO_4_ was added so that the N/P ratio of the overlying water was 14.93, which was similar to the annual average ratio in Taihu Lake during 2007 (14.49). The nutrient concentrations for the experiments were selected according to the nutrient levels of the eutrophic Lake Taihu in China, and the highest nitrate concentration was based on the maximum TN of Taihu in 2007 (20.79 mg N l^−1^).

The bottles were left in the field for 28 and 31 days, respectively, to allow airborne organisms (for example, bacteria) to colonize the water and sediments of microcosms. The field setups were completed in 3 days. To keep the species dispersal events as natural as possible, we did not cover the experimental set-ups in case of rainfall. We checked the experimental set-ups twice during each experimental period, and added sterilized MilliQ water to obtain a final volume of approximately 1.2 l. Filling to a volume of 1.2 l with artificial freshwater into the 1.5 l bottles ensured that the water would not overflow due to rain or splash out in the heavy rains during the experimental periods.

To avoid the effects of daily temperature variation, we measured the water temperature and pH within 2 h before noon at all elevations in the day before the final sample collection. At the end of the experimental period, we aseptically sampled the water and sediments of each bottle. The samples were frozen at −20 °C after sampling until chemical and molecular analyses.

It should be noted that we analysed the sediment bacteria, but not the water column bacteria. We did not use any specific natural aquatic bacterial communities from ponds or lakes in the current experiments, but established new communities via post-dispersal effects. More details on the experimental design are provided in the Supplementary Methods.

### Physicochemical and biological analyses

Water ammonium (NH_4_^+^), nitrate (NO_3_^−^), nitrite (NO_2_^−^) and dissolved inorganic phosphorus (PO_4_^3−^) were measured with a flow injection analyser (Skalar SA1000, Breda, Netherlands). Sediment Chl *a* was extracted with 90% acetone^49^. Sediment genomic DNA was extracted using the phenol chloroform method, and bacterial 16S rRNA genes were amplified in triplicate using universal bacterial primers^28^. Real-time qPCR quantification of bacterial 16S rRNA genes was performed on an iCycler iQ5 thermocycler (Bio-Rad, Hercules, CA) as described previously^60^. PCR products were sequenced with MiSeq (Illumina, San Diego, CA). The sequences were processed in QIIME (v1.8)^28^,^61^. OTUs were defined at 97% sequence similarity. The bacterial sequences were rarefied to 18,000 per sample. More details are provided in the Supplementary Methods.

### Statistical analyses

Non-metric multidimensional scaling (nMDS) was based on the community Sørensen similarity, which is a popular beta diversity metric used in ecological studies for DDRs^31^,^62^ and was applied in a general framework for the distance-decay of similarity in ecological communities^63^. To test the hypothesis that region and elevation structure the bacterial communities, PERMANOVA was used^64^. To identify important environmental factors related to communities, we performed Mantel tests (permutations=9,999) on the community Sørensen similarity, Pearson correlations using the first axis of nMDS, and a canonical correspondence analysis on species abundance data.

We used linear and quadratic models to explore the relationships between alpha and gamma diversity with elevation and nutrient. The more appropriate model was selected based on a lower value of Akaike's information criterion^65^, and F-statistic was used to test the significance of regression. We used Pearson correlations to explore the relationships between species richness and environmental variables. We also applied stepwise multiple regression analyses with forward selection of variables to identify the most important environmental factors explaining community composition (that is, sample scores on the first axis of nMDS) and species richness.

Water temperature was highly correlated with elevation, and was among the strongest factors related to species richness and community composition (Supplementary Fig. 6); therefore, we used water temperature to explore the relationships between temperature and species richness or composition. For each region, we fit linear and quadratic models for the TRR of each nutrient level and found the quadratic model to be better in 7 out of 20 cases. However, significant linear models also fit well in 15 out of 20 cases (Supplementary Fig. 9). Thus, we examined the TRR with the MTE, where the lognormal richness is a linear function of temperature, expressed as 1/*kT*, in which *k* is Boltzmann's constant and *T* is absolute temperature in K. The slope of TRR was defined as the activation energy (*E*), indicating the temperature dependence of species richness. Furthermore, the slopes of the temperature DDR, based on Sørensen similarity, were used to explore the turnover rates of species composition across temperature gradients. Finally, the slopes of TRR and temperature DDR were related to nutrient enrichment and the relationships were explored with linear and quadratic models. The better model was selected based on lower value of Akaike's information criterion. For NRR, the slope of the linear regression was used to represent changes in species richness with nutrient enrichment (log_10_). The slopes of nutrient DDR were used to investigate the turnover rates of species composition across nutrient gradients. The significance of DDR slopes was tested with Mantel test (permutations=9,999).

We explored the relationships between temperature, nutrient enrichment, and bacterial communities using PLS-PM in the R package plspm (V0.4.7)^47^. This method is known as the partial least squares approach to structural equation modelling and allows for the estimation of complex cause-effect relationship models with latent variables^47^, which was especially suitable for our experimental data with strong environmental gradients. Five latent variables were used: temperature (the measured water temperature and its squared value), nutrient enrichment (the initially added nutrients and measured nutrients), primary productivity (Chl *a* and pH), diversity (species richness), and composition (the first axis of nMDS). We used pH as a proxy for primary productivity because of its positive correlations with Chl *a* (Supplementary Fig. 5). Observed variables were selected based on collinearity and prediction power for diversity and composition. Most of the loadings for observed variables on latent variables were>0.7 (Supplementary Fig. 11). We ran PLS-PM using 1,000 bootstraps to validate the estimates of path coefficients and the coefficients of determination^47^. Path coefficients represent the direction and strength of the linear relationships between variables, or the direct effects. Indirect effects are the multiplied path coefficients between a predictor and a response variable, adding the product of all possible paths excluding the direct effect. Models with different structures were evaluated using the goodness of fit statistic^47^.

### Data availability

The amplicon sequences were deposited in MG-RAST under accession number 17710. Other relevant data in this study are available from the authors.

## Additional information

**How to cite this article:** Wang, J. *et al*. Nutrient enrichment modifies temperature-biodiversity relationships in large-scale field experiments. *Nat. Commun.* **7,** 13960 doi: 10.1038/ncomms13960 (2016).

**Publisher's note:** Springer Nature remains neutral with regard to jurisdictional claims in published maps and institutional affiliations.

## Supplementary Material

Supplementary Information Supplementary Figures, Supplementary Tables, Supplementary Methods and Supplementary References.
