# Supplementary material for: Nutrient enrichment modifies temperature-biodiversity relationships in large-scale field experiments
Source: Nat Commun. 2016 Dec 21;7:13960. doi: 10.1038/ncomms13960 (PMC5187590; doi:10.1038/ncomms13960)
Supplement: Supplementary Information — Supplementary Figures, Supplementary Tables, Supplementary Methods and Supplementary References. [file ncomms13960-s1.pdf]

Supplementary Methods

Experimental design

We analysed the sediment bacteria, but not the water column bacteria, for the following five reasons. Firstly, the natural aquatic systems are mostly composed of sediment and water; thus, the sediment is an essential part of aquatic microcosms. By adding both sediment and artificial lake water, we experimentally assembled whole, natural aquatic environments. Secondly, sediment acts as a minimal medium with all the necessary nutrients and mineral elements for the growth of microorganisms that are not contained in the artificial lake water. Thirdly, we expected that the sediment bacteria would be less influenced by disturbance effects because the sediment bacteria were in more stable physical conditions than the water column bacteria. The sediment was below the ground surface, and thus the sediment bacteria were less exposed to ambient environmental variations, such as UV radiation, than the bacteria in the water column. Sediment bacteria may also face less grazing pressure from higher organisms than water column bacteria, which are prone to grazing. Fourthly, we expected that the sediment bacteria were less structured by stochastic processes because they were environmentally selected, first by water column and then by sediment. Our previous study showed that deterministic processes are more important in structuring the bacterial communities in sediments than of the overlying water within aquatic environments [1]. Finally, compared to water column bacteria, especially those with low nutrient supply and thus with low biomass, sediment bacteria were much easier to collect after siphoning out the overlying water, preserve in sterile tubes in the field conditions, and examine with molecular methods. We faced practical issues with sampling the bacteria in the overlying water

because of the very large nutrient gradients. For example, the bacterial biomass in the overlying water at the lowest nutrient treatment (e.g., controls) was too low for the DNA analyses with the small volume of overlying water left over after the chemical analyses. Furthermore, we considered a large number of samples from each mountain, and the logistical issues thus prevented us from efficiently sampling the water-column bacteria. Due to the remote field conditions, we could not manually the filter water samples in situ for bacteria within a reasonable time period, and it was not possible to carry the water back to the laboratory. Even if the water had been transferred to the laboratory, the experimental delay (e.g., in days) would likely to happen for additional water-filtering manipulations.

In the one-month-incubation experiments, the sediment bacterial species were selected by two environmental filters, the overlying water and sediments. The simplified experimental ecosystems were highly suitable for the examination of the relative importance of temperature and nutrient factors on bacterial communities. Because bacterial species are mainly dispersed through the air, the colonized communities allowed us to capture the most natural local or regional species pools possible. Due to the multiple dispersal sources of bacterial species at local, regional, and global scales [2, 3], we could not identify the exact origins of sediment bacteria in the microcosms. This challenge was especially prevalent after the bacteria in the sediment were further filtered by the local environments of the overlying water and sediment in the microcosms. Thus, we did not examine bacterial communities of the samples from other local habitats, such as air dust, soil, streams, and lakes, to track the sources of sediment bacteria in the microcosms.

We did not use any specific natural aquatic bacterial communities from ponds or lakes in the current experiments, but established new communities via post-dispersal effects. By using sterile microcosms, we simplified the underlying processes for the observed bacterial communities by partly eliminating the legacies of historical events that affect the communities in natural ponds or lakes. This is because, together with contemporary environmental filtering, historical contingencies are one of the main forces affecting present-day microbial communities [4]. Compared to the microcosms, natural aquatic environments such as ponds or lakes are influenced by numerous covariant variables that are difficult to control for but that are important for organisms [5, 6, 7]. (1) The origin of natural aquatic environments affects the evolution and dispersal history of species in their biological communities. For instance, the selected lakes might have different origin and formation histories that affect their morphology and hydrological conditions. (2) Catchment area, surrounding vegetation, and local physical-chemical conditions also vary spatially and affect the aquatic biota. (3) The initial communities based on natural communities of ponds or lakes can differ spatially thus resulting in very heterogeneous initial communities, and the biological interactions with other organisms are probably more complex in nature than in our experimental microcosms. (4) To have consistent initial communities by inoculating microcosms with natural communities of ponds or lakes (i.e., the sediment bacteria in Taihu Lake) is less feasible for such broad-scale experiments due to the difficulties in preserving and transporting these natural aquatic communities for large distances and long periods of time. (5) Most importantly, using natural communities from ponds or lakes as the initial community does not eliminate the dispersal effects from the air, and thus the potential for strong priority

effects [8] that would likely occur. This is because the air is one of the main dispersal routes for bacterial species in nearly all surface aquatic environments [9, 10, 11].

Physicochemical analyses

The concentrations of ammonium (NH_4^+), nitrate (NO_3^-), nitrite (NO_2^-), and dissolved inorganic phosphorus (PO_4^{3-}) in the overlying water were measured with a flow injection analyser (Skalar SA1000, Breda, Netherlands). The sediment total organic carbon (TOC) was determined using a solid TOC analyser (SSM-5000A, Shimadzu, Japan). The sterilized sediments before the field experiments were measured for the above variables in three replicates.

We did not measure all the potentially important variables for the bacterial communities in the sediment, such as oxygen concentration or UV radiation. This is largely because we have no evidence about the potential importance of these variables on sediment microbial communities. Further, we do not expect these potential effects to be stronger than nutrient and temperature effects considered in our study. This is because the bottles were buried in the local soil to approximately 10% of the total bottle height, and the sediment in each bottle was below the ground surface. Therefore, it is unlikely that UV radiation would have had strong effects on these communities that would mask the temperature or nutrient effects on the bacteria. In addition, we added 15 g sterilized sediment to the bottom of each bottle, which created a sediment layer of approximately 5 mm. Based on our previous studies on the oxygen concentrations at the sediment-water interface in eutrophic lakes, e.g., Wang et al. [12, 13], the 5-mm sediment layer would be less likely to be anoxic during the experimental period, especially with the shallow overlying

water column (approximately 18 cm) and low total organic carbon (0.587 ± 0.390%).

Bacterial community analyses

Genomic DNA was extracted from freeze-dried sediments using the phenol chloroform method [14]. Real-time qPCR quantification of bacterial 16S rRNA genes in sediments was performed on an iCycler iQ5 thermocycler (Bio-Rad, Hercules, USA) as described previously [15]. Bacterial 16S rRNA genes were sequenced using MiSeq (Illumina, San Diego, USA) as described previously [16]. We amplified 16S rRNA genes in triplicate using the universal bacterial primers 515F, 5'-GTGCCAGCMGCCGCGGTAA-3' and 806R, 5'-GGACTACHVGGGTWTCTAAT-3', targeting the V4 region. Positive PCR products were confirmed with agarose gel electrophoresis. PCR products from triplicate reactions were combined and quantified with PicoGreen. PCR products from samples sequenced in the same MiSeq run were pooled at equal molality. The pooled mixture was purified with a QIAquick Gel Extraction Kit (QIAGEN Sciences, Germantown, USA) and re-quantified with PicoGreen. Sample libraries were prepared according to the MiSeqTM Reagent Kit Preparation Guide (Illumina, San Diego, USA).

We processed the sequences primarily using the QIIME pipeline (v1.8) [17] following recent references e.g., [16, 18]. Briefly, overlapped paired-end sequences from MiSeq were assembled using FLASH [19] and poorly overlapped and poor quality sequences were filtered out before de-multiplexing based on barcodes. Then, the sequences were clustered into OTUs at 97% pairwise identity with the seed-based uclust algorithm [20]. After chimeras were removed via Uchime, and representative sequences from each OTU were aligned to the Greengenes imputed core reference alignment V.201308 [21] using

PyNAST [22]. The taxonomic identity of each representative sequence was determined using the RDP Classifier [23] and chloroplasts were removed.

We removed singletons before the subsequent analyses, which is a common procedure in microbial studies, and has been highly recommended in popular microbial analysis pipelines even before the generation of OTU tables (Supplementary Fig. 12). We found that the removal of singletons and rare species (e.g., those occurring in less than 6 samples) did not notably affect the patterns in species richness in our data (Supplementary Fig. 12). To keep the reported results as simple as possible, we used the community OTU table without singletons for all analyses. For Archaea, we obtained 102 sequences from 42 out of 300 total samples. These archaeal sequences can be classified into 13 OTUs related to the families Nitrososphaeraceae, Nitrosocaldaceae, Methanocellaceae, Methanobacteriaceae, Methanomicrobiaceae, Methanosaetaceae, Sulfolobaceae, and DHVEG-1. Due to the insufficient sequence number for Archaea, we excluded all archaeal sequences from the data.

Supplementary Tables

Supplementary Table 1. Regression analyses of biodiversity with environmental variables. Multiple regression analyses of bacteria diversity and community composition were performed as a function of environmental variables. For diversity and community composition, species richness and the first axis of non-metric multidimensional scaling, respectively, were used as the response variables. The best models were identified using Akaike's information criterion, and variables were selected in multiple regressions only if *P* < 0.05. All of the variables were standardized (mean = 0; SD = 1) and are displayed with increasing *P*-values. Temp: measured water temperature. Temp.2: the squared value of measured water temperature. ADD.NO3: the initially added NO_3^-. NO3, NO2, and pH: water NO_3^- and NO_2^-, and pH, respectively. Chl *a*: sediment Chl *a* (mg·g^{-1}). Copies: copy number of 16S rRNA genes qualified by real-time qPCR (copy number per gram dry sediment).

Response	Mountains	Model r^2	Explanatory variables and Beta-weights$^{\$}$					
Diversity	Norway	0.595	Temp ***	Temp.2 ***	NO2 ***	pH *		
Diversity	China	0.397	Temp ***	Temp.2 ***	NO2 ***	Chl *a* *		
Composition	Norway	0.605	Temp ***	Temp.2 ***	NO2 ***	pH ***		
Composition	China	0.880	Temp.2 ***	pH ***	NH4 ***	NO3 **	ADD.NO3 *	Copies *

$^{\$}$ Standardized partial regression coefficients, ****P* < 0.001; ***P* < 0.01; **P* < 0.05.

Supplementary Figures

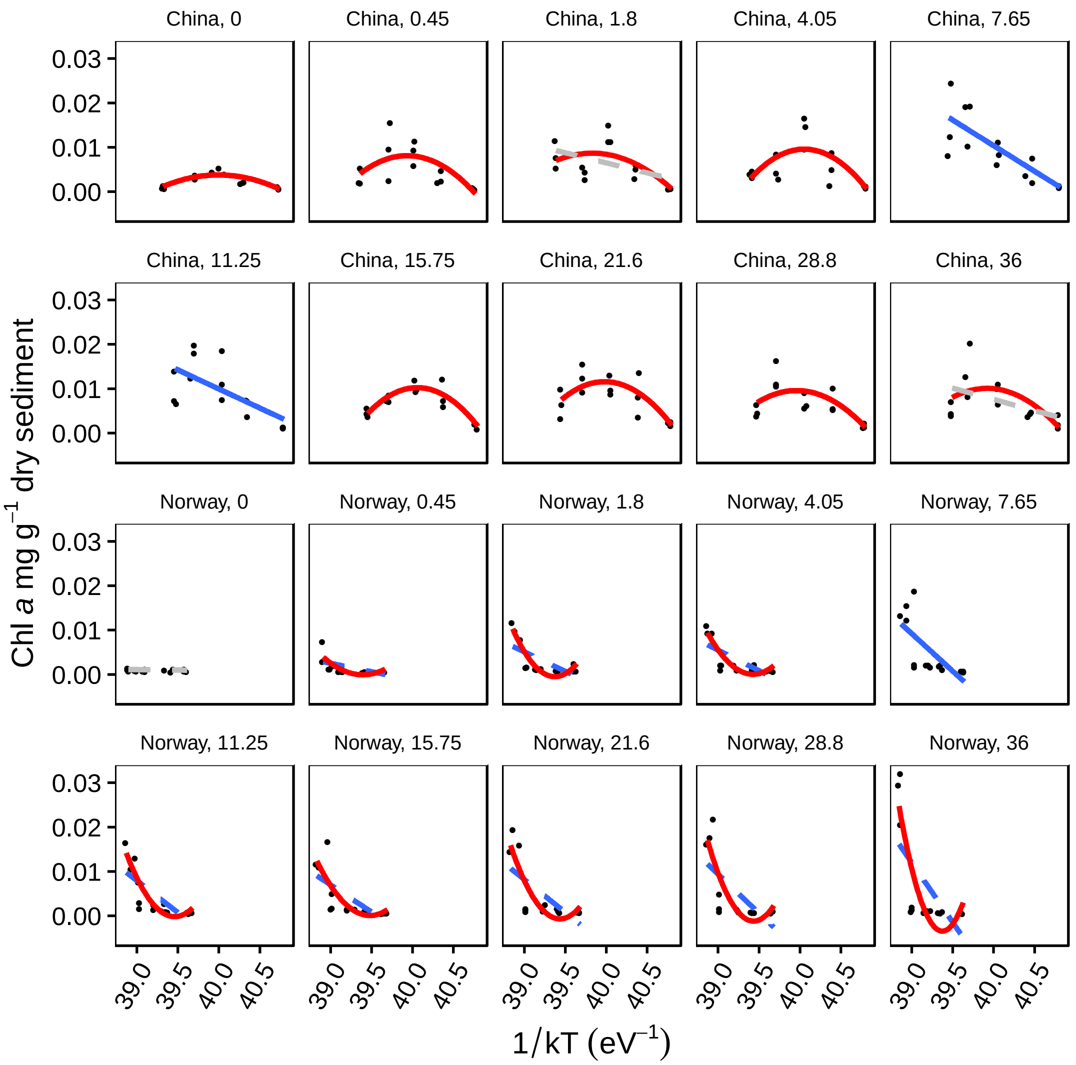

Supplementary Figure 1. The relationships between sediment Chlorophyll *a* and temperature. The relationships were analyzed for each nutrient enrichment level and location, and we used the inverse absolute temperature (1/kT), where k is Boltzman's constant 8.62 × 10 $^{-5}$ eV K $^{-1}$, and T is absolute temperature in Kelvin. These relationships are fitted by linear and quadratic models, the significance of which ($P < 0.05$, F-test) are shown with blue and red lines (solid or dotted). The better model, shown in solid line, was selected based on lower value of Akaike's information criterion. Gray dotted lines indicate non-significant relationships of a linear model ($P > 0.05$, F-test).

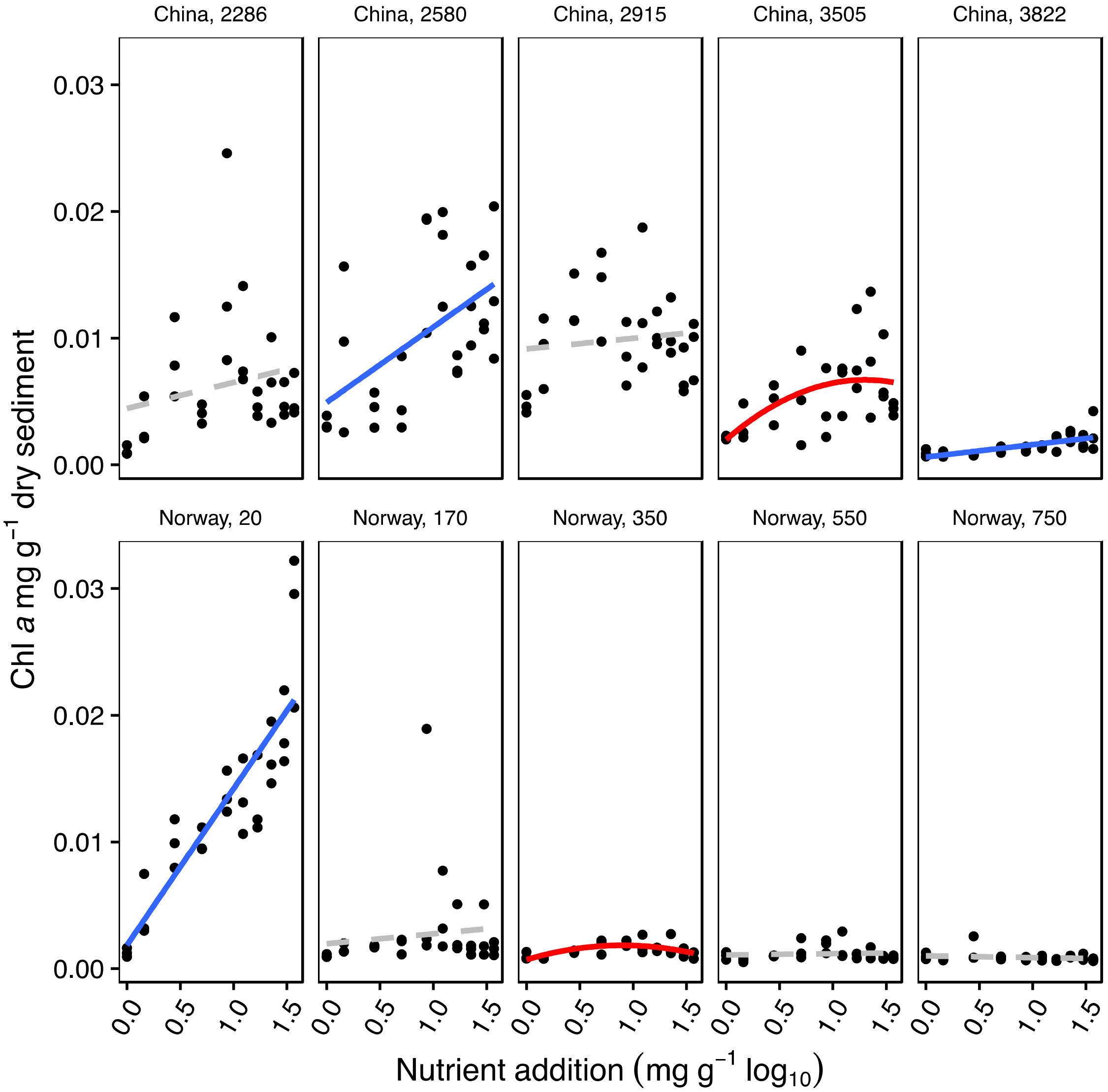

Supplementary Figure 2. The relationships between sediment Chlorophyll *a* and nutrient enrichment. These relationships for each elevation band were fitted by linear and quadratic models, the significance of which ($P < 0.05$, F-test) are shown with blue and red lines (solid or dotted). The better model, shown in solid line, was selected based on lower value of Akaike's information criterion. Gray dotted lines indicate non-significant relationships of a linear model ($P > 0.05$, F-test).

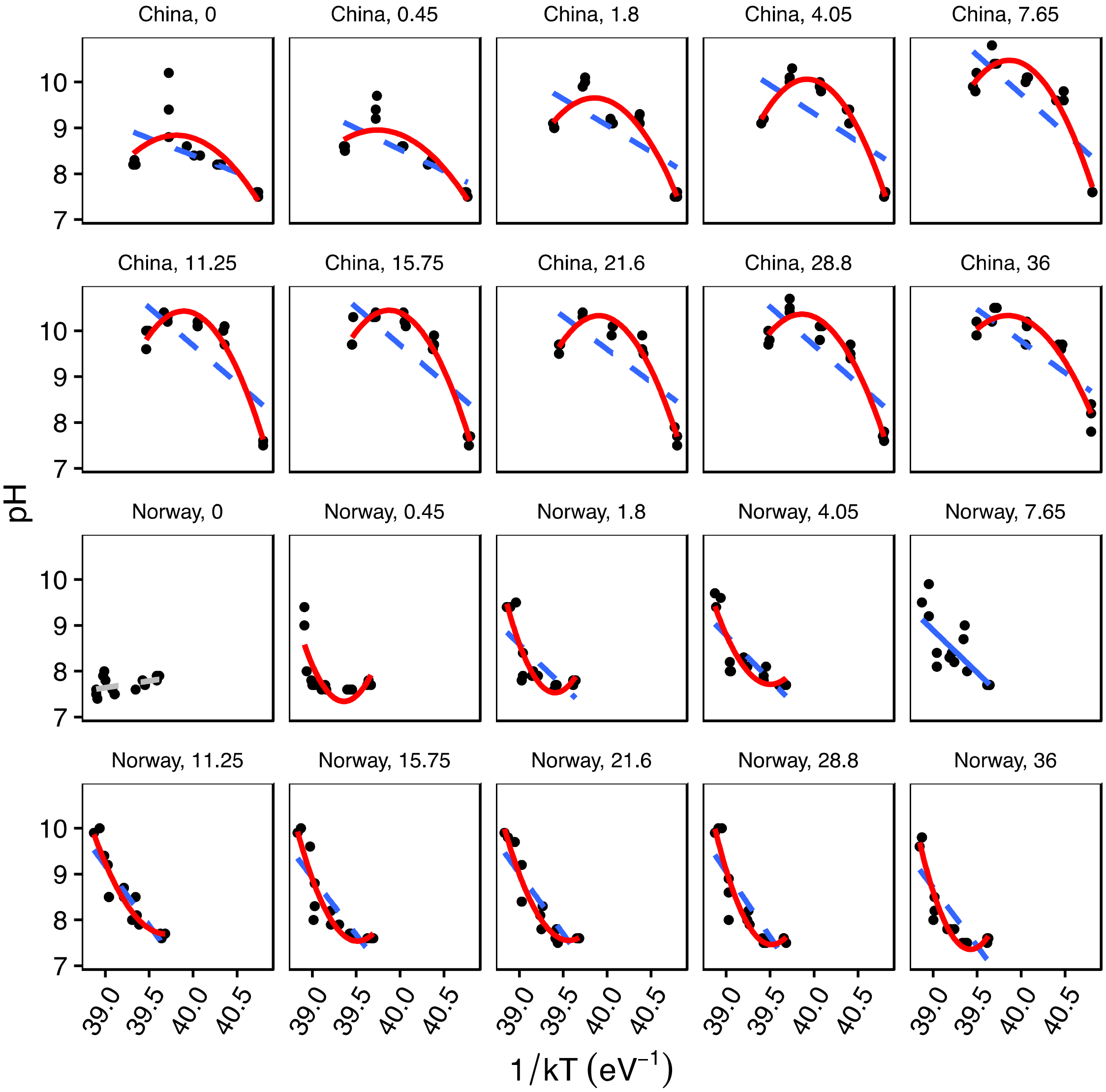

Supplementary Figure 3. The relationships between overlying water pH and temperature. These relationships for each nutrient enrichment and location were fitted by linear and quadratic models, the significance of which *(P* < 0.05, F-test) are shown with blue and red lines (solid or dotted). The better model, shown in solid line, was selected based on lower value of Akaike's information criterion. Gray dotted lines indicate non-significant relationships of a linear model (*P* > 0.05, F-test).

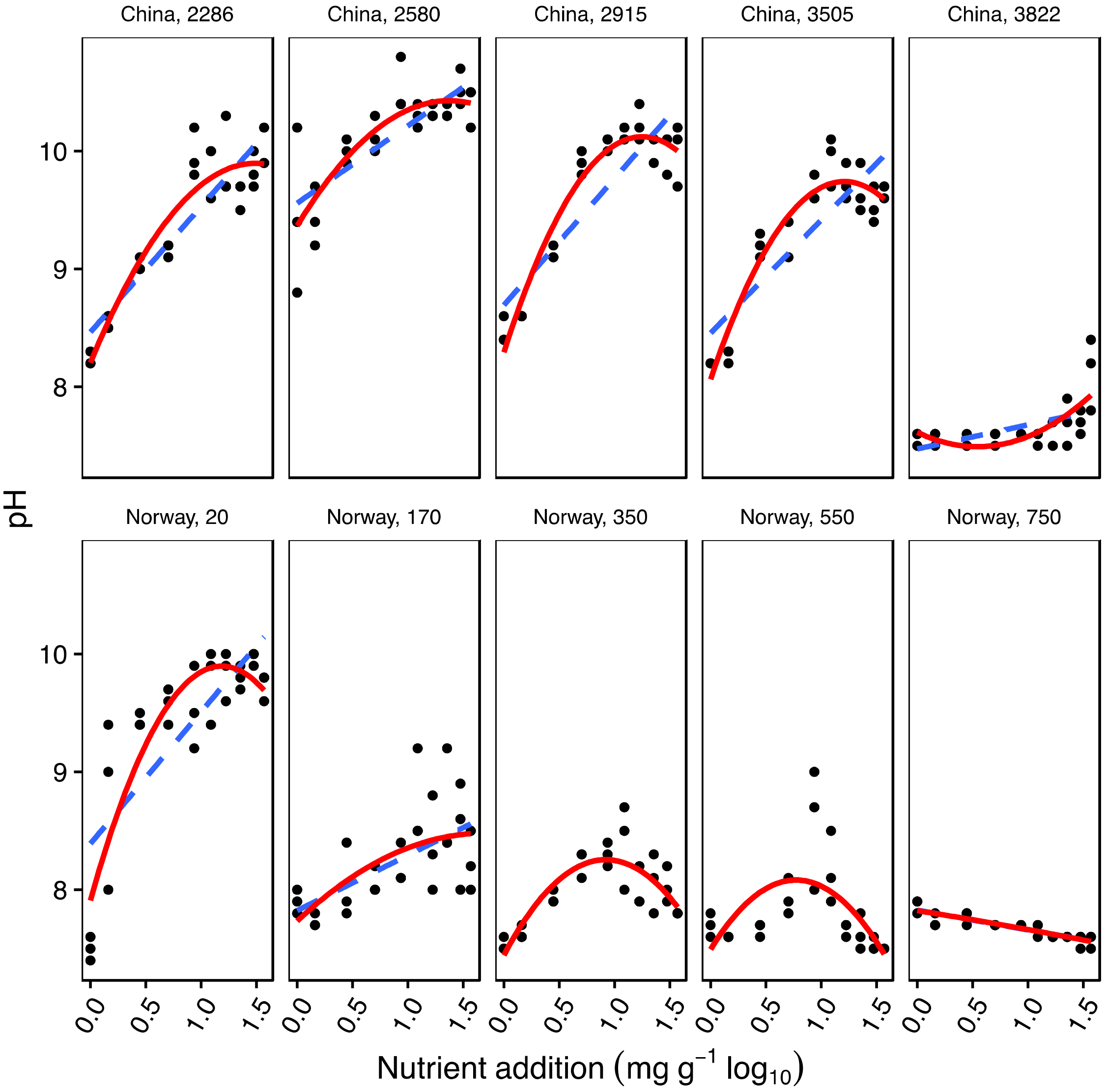

Supplementary Figure 4. The relationships between overlying water pH and nutrient enrichment. These relationships for each elevation band were fitted by linear and quadratic models, the significance of which ($P < 0.05$, F-test) are shown with blue and red lines (solid or dotted). The better model, shown in solid line, was selected based on lower value of Akaike's information criterion. Gray dotted lines indicate non-significant relationships of a linear model ($P > 0.05$, F-test).

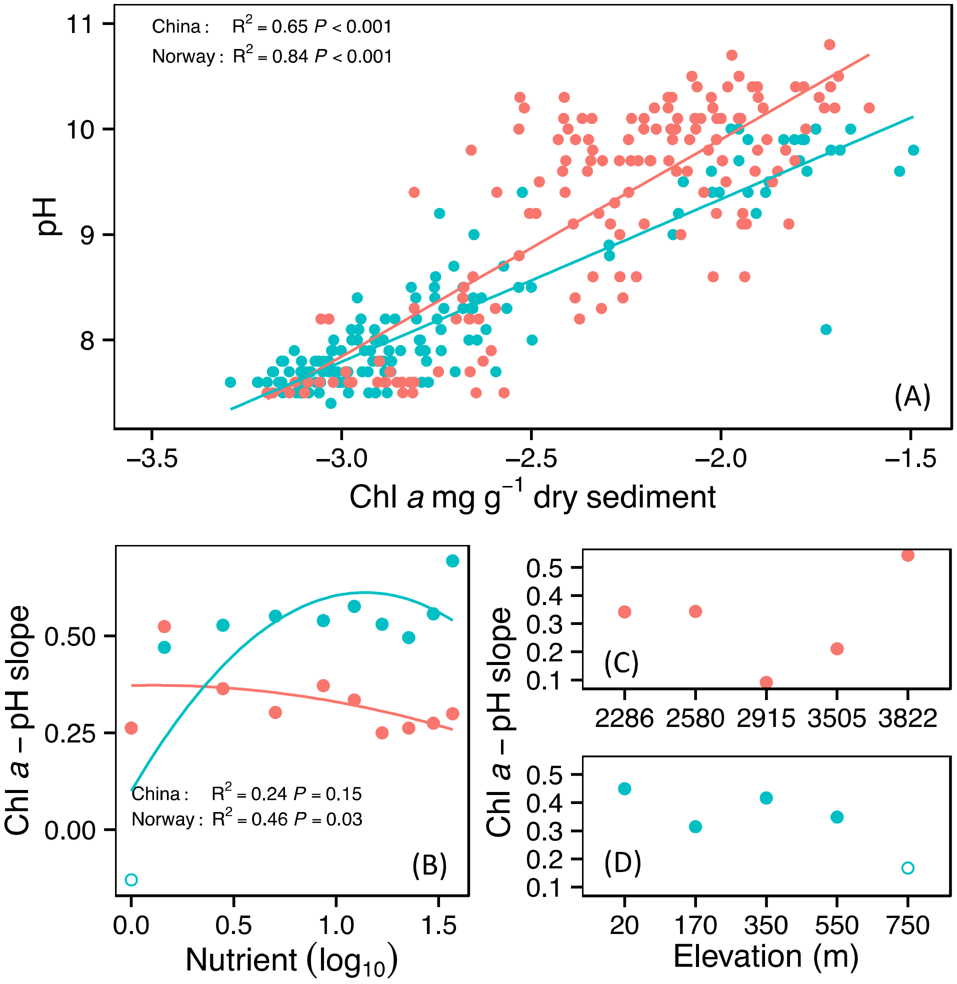

Supplementary Figure 5. The relationships between sediment Chlorophyll *a* and overlying water pH. The relationships were fitted with linear models and significances were examined with F-test. (A) The linear relationship between Chlorophyll *a* and pH for each region. (B) The slopes of Chlorophyll *a*-pH relationships along nutrient enrichment. (C, D) The Chlorophyll *a*-pH relationships along elevational gradients. Solid dots indicate the significant ($P < 0.05$, F-test) relationships (B-D). Red colour for China and blue colour for Norway.

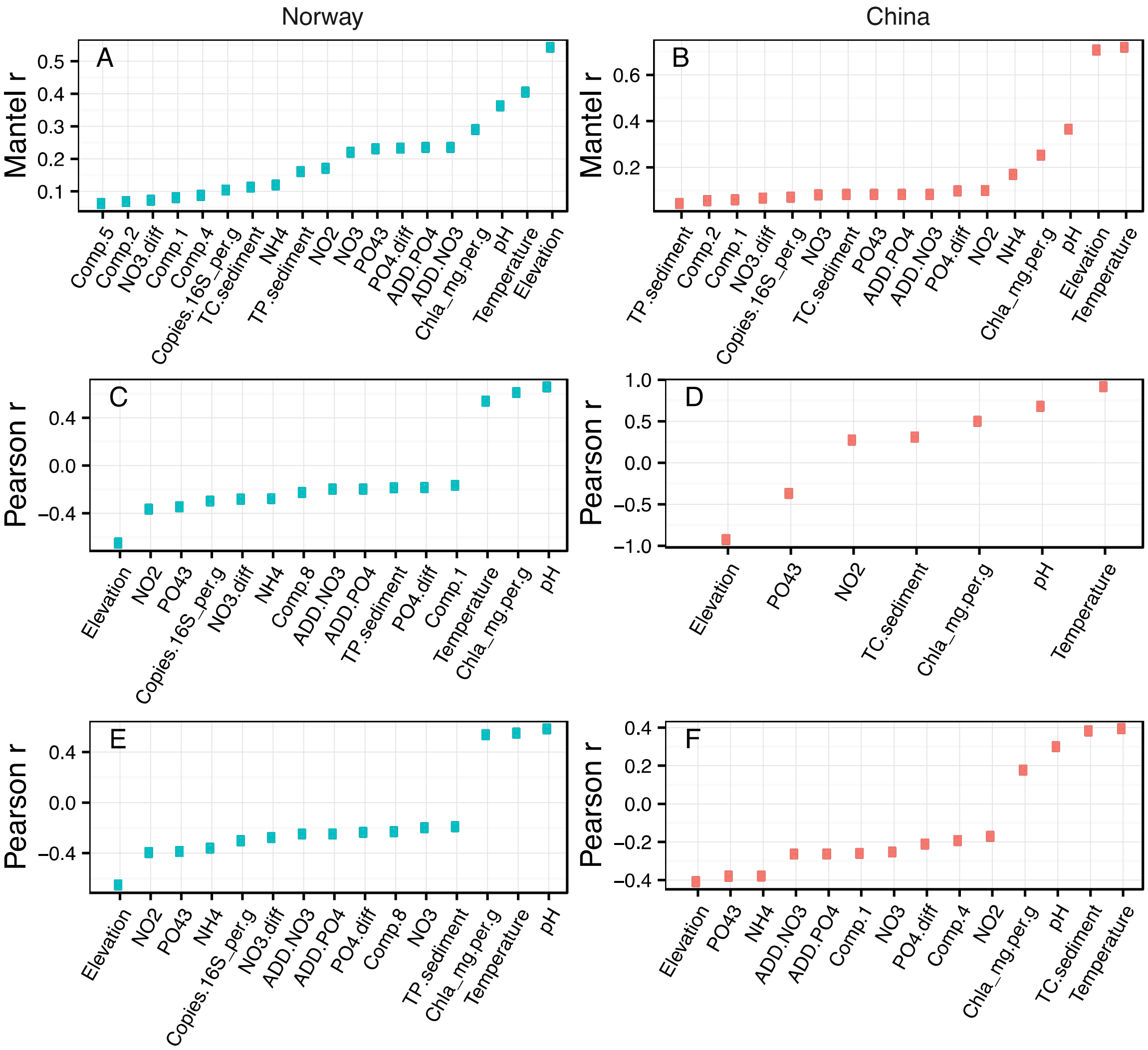

Supplementary Figure 6. Correlations between environmental variables and bacterial biodiversity. We only included the significant ($P < 0.05$) environmental variables related to community similarity based on Mantel test (A, B), the first axis of non-metric multidimensional scaling of the communities with Pearson correlations (C, D) and species richness with Pearson correlations (E, F). Temperature: Water temperature in situ. pH: Water pH. Chla_mg.per.g: Chl *a* in the sediments ($mg \cdot g^{-1}$). NH4, NO2, and NO3: NH_4^+, NO_2^-, and NO_3^- in the water at the end of experiments. ADD.NO3 and ADD.PO4: initial NO_3^- and PO_4^{3+} added to the microcosms. NO3.diff and PO4.diff: NO_3^- and PO_4^{3+} differences between the start and end of the experiments. TC.sediment and TP.sediment: total organic carbon and total phosphorus in the sediments at the end of experiments. Copies.16S_per.g: copy number of 16S rRNA genes qualified by real-time qPCR (copy number per gram dry sediments). Comp.1, Comp.2, Comp.3, Comp.4 and Comp.5: principal components of metal concentrations (such as K, Na, Ca, Mg, Al, Fe, Zn, Cu, Cr and Ni).

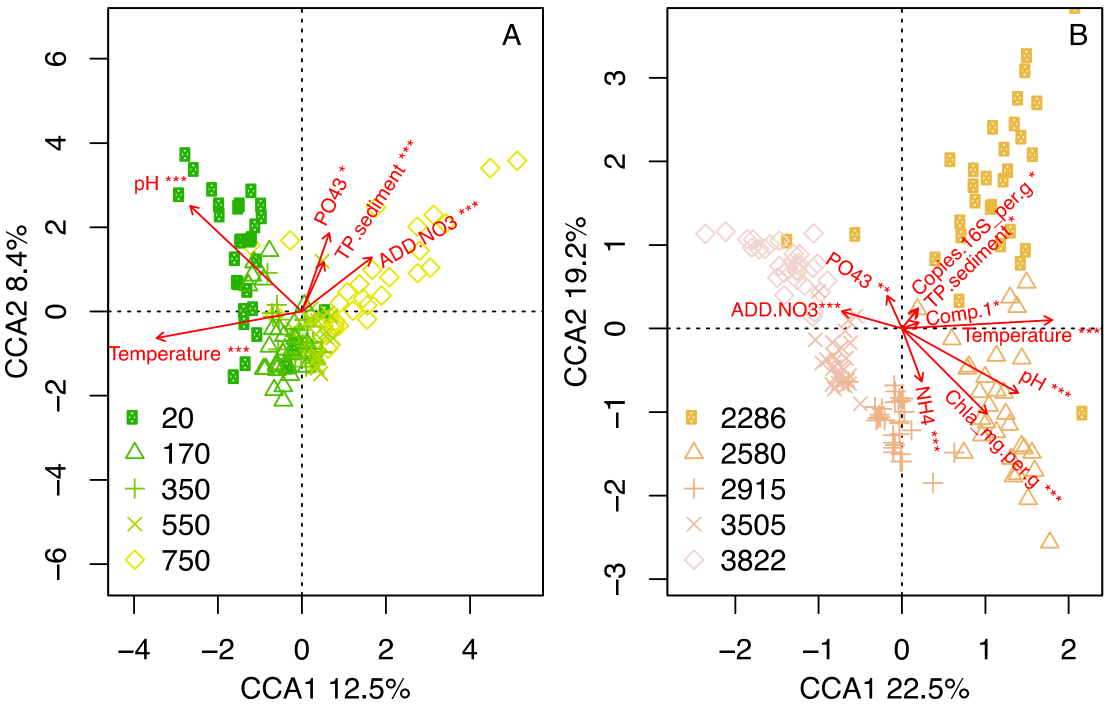

Supplementary Figure 7. Canonical correspondence analysis for the bacterial communities. A is for Norway, and B is for China. Detrended correspondence analysis was used to determine that the gradient length of the species abundance matrix is larger than 2.0 along the first axis, thus implying a unimodal species responses. Thus, considering the long environmental gradients (especially for nutrients), we applied canonical correspondence analysis (CCA) to examine the relationships between bacterial community compsition and explanatory variables. All environmental variables, except pH, were log-transformed [by log(X + 1) or log(1000X + 1)]. The abbreviations of environmental variables are given in Supplementary Figure 6. The elevations (units: m a.s.l) were shown with different colors. The significance of variables was assessed with permutation test (n = 1,000). *, $P < 0.05$; **, $P < 0.01$; ***, $P < 0.001$.

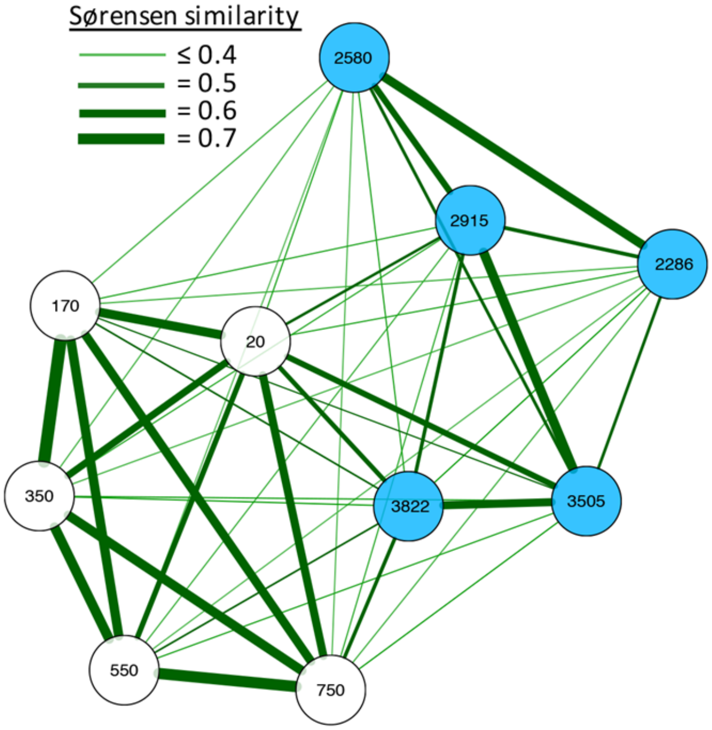

Supplementary Figure 8. The community Sørensen similarity between elevations of the two mountains. Blue: The Laojun Mountain, China. White: The Balggesvarri Mountain, Norway. The elevations (unit: m a.s.l.) of each site are shown in the nodes. Thicker edges indicate high Sørensen similarity in bacterial composition between elevations within and between regions.

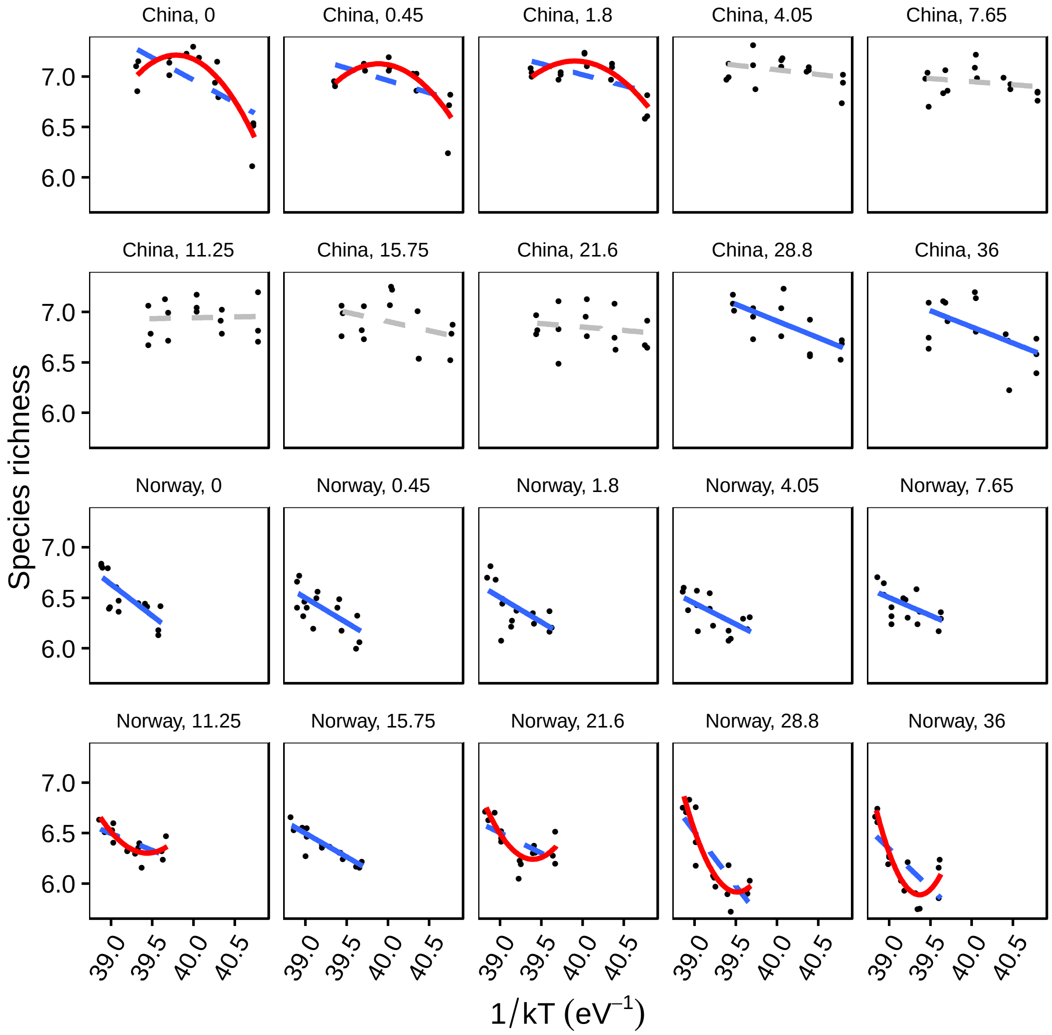

Supplementary Figure 9. The relationships between temperature and bacterial species richness. These relationships for each nutrient enrichment and location were fitted by linear and quadratic models, the significance of which ($P < 0.05$, F-test) are shown with blue and red lines (solid or dotted). Species richness was log-transformed, and we used the inverse absolute temperature (1/kT), where k is Boltzman's constant 8.62×10^{-5} eV K^{-1}, and T is absolute temperature in Kelvin. The better model, shown in solid line, was selected based on lower value of Akaike's information criterion. Gray dotted lines indicate non-significant relationships of a linear model ($P > 0.05$, F-test).

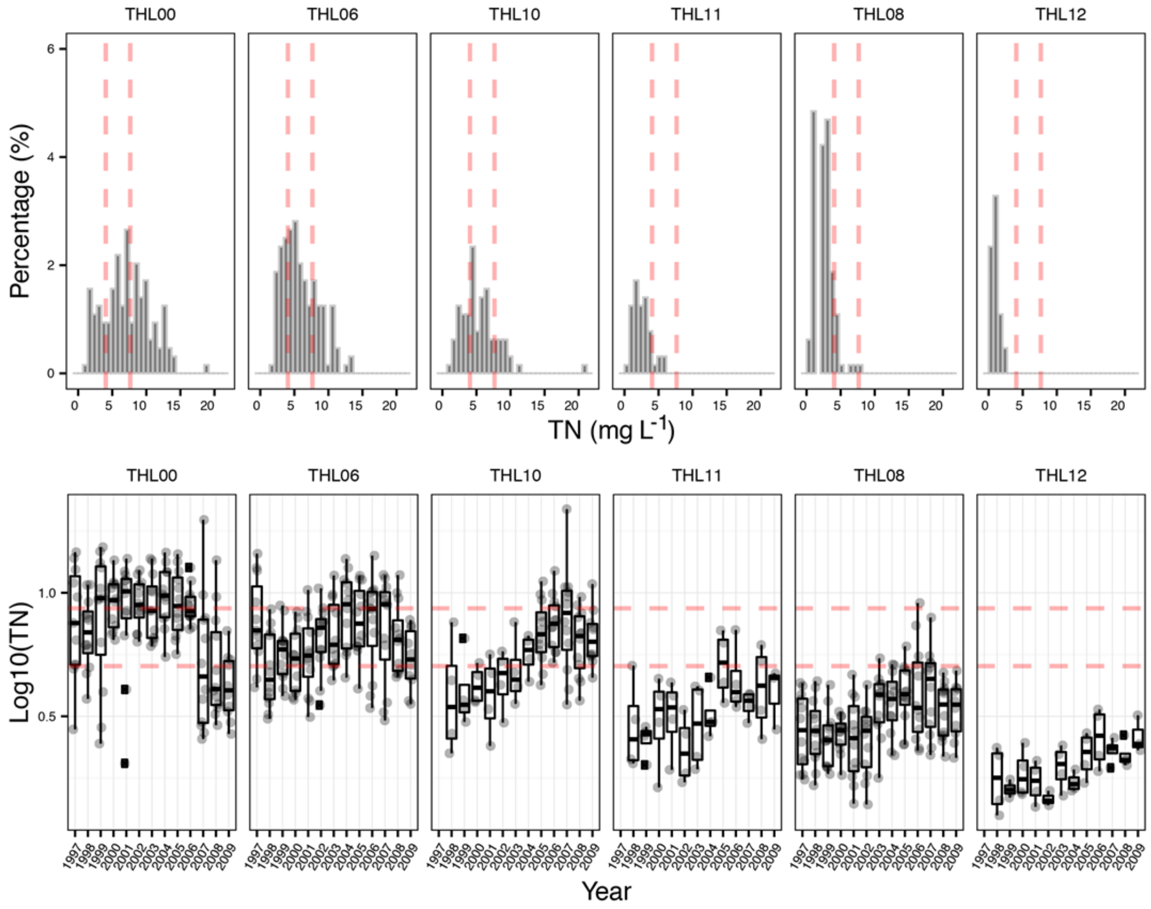

Supplementary Figure 10. The nutrient concentrations in Taihu Lake, China, in the years 1997-2015. We considered total nitrogen (TN), which was measured monthly by CNERN, Taihu Laboratory for Lake Ecosystem Research. We selected six monitoring sites from river mouth (THL00, THL06, THL10, and THL11), lake center (THL08), and macrophyte-dominated region (THL12), which present clear nutrient variations geographically. Upper panel: The relative frequency histogram of TN concentrations for each site. Lower panel: The boxplot of TN through the years for each site. The red dotted lines indicate the intermediate nutrient enrichment observed in this study: 4.05 and 7.65 mg N L^{-1} NO_3^-, respectively. These results showed that observed TN concentrations in Taihu Lake, exhibiting a high spatial heterogeneity within the lake, are frequently higher than the intermediate nutrient enrichment values in our experiments (i.e., ~4.05-7.65 mg N L^{-1} NO_3^-).

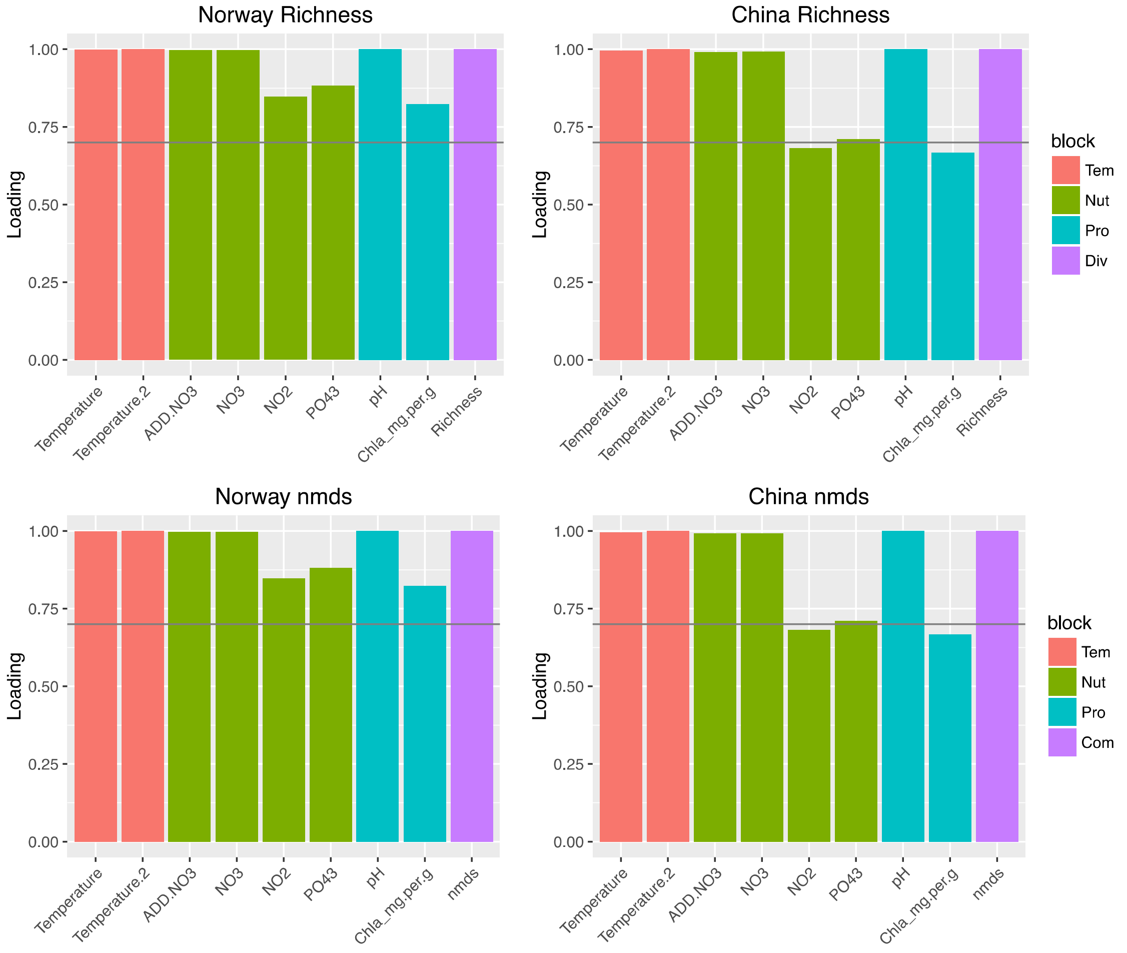

Supplementary Figure 11. The loadings of observed variables for the latent variables in path models. The observed variables were used for the partial least squares path modeling (Fig. 4), and we showed their loadings for the latent variables. Most of the loadings were selected with the threshold 0.7, as suggested in previous studies [24]. The observed variables included for each latent variable were shown in difference colors. There latent variables are Temperature (Tem), Nutrient (Nut), Productivity (Pro) and Diversity (Div) or Community composition (Com). Temperature: measured water temperature. Temperature.2: the squared value of measured water temperature. ADD.NO3: the initially added NO_3^-. NO3, NO2, and pH: water NO_3^- and NO_2^-, and pH, respectively. Chla_mg.per.g: sediment Chl *a* ($mg{\cdot}g^{-1}$).

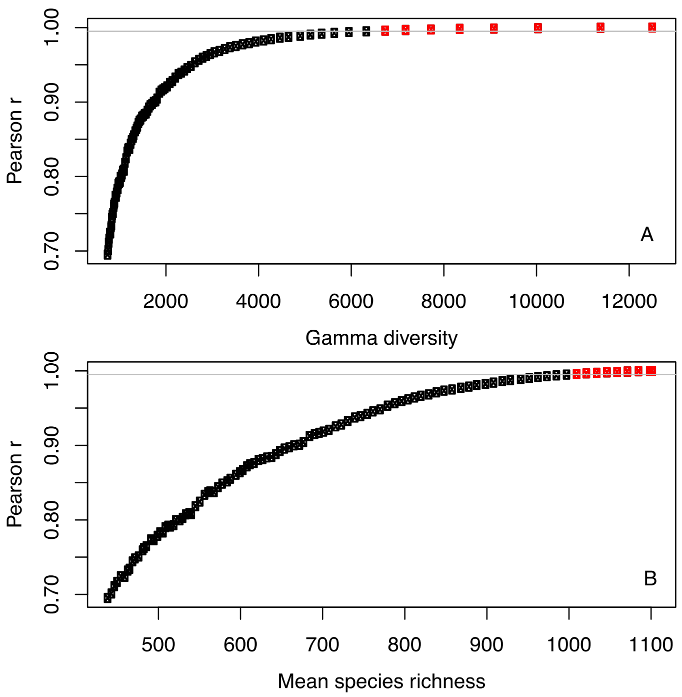

Supplementary Figure 12. The effects of removal of singleton or other rare species on species richness.

We removed the singletons or other rare species from the full community data set (that is, with singletons and 300 total samples). Singleton was defined as a read with a sequence that is present exactly once, i.e. is unique among the all sequence reads. The rare species was defined continuously as the species occuring in only 1 sample, 2, 3, …. , or 100 samples. For each community with species removal, gamma diversity (i.e., x-axis in panel A) is the total OTU number of full data set, and mean species richness (i.e., x-axis in panel B) is the average species richness of the 300 samples. Gamma diversity and mean species richness were highest with singletons included, followed by the removal of the rare species (i.e., occuring in only 1 sample, 2, 3, …. , or 100 samples).

For the obtained diversity (A: gamma diversity; B: mean species richness), we calculated the Pearson correlations (i.e., y-axis in panels A and B) between diversity of the communities without singletons and that of the communities with singletons or the communities without rare species. All Pearson correlations were significant ($P < 0.01$), and Pearson r decreased continously along the gradients of both gamma diversity (A) and mean species richness (B). For the community data (in red color) with the removal of singletons and rare species occuring in less than 6 samples, the Pearson correlations were quite similar, and all higher than 0.995, indicated by gray horizontal lines (A, B).

These results showed that the effects of OTU removal on the values of species richness were low when the singletons and other rare species were excluded. Furthermore, it is common in microbial studies to remove singletons before detailed ecological analyses, and it is also highly recommended in microbial analysis pipelines (such as usearch, http://www.drive5.com/usearch/manual/singletons.html; Qiime, http://qiime.org/tutorials/open_reference_illumina_processing.html). Thus, to keep our reported results as simple as possible, we finally used the OTU tables of bacterial community without singletons.

Supplementary References

1. Wang J, *et al.* Phylogenetic beta diversity in bacterial assemblages across ecosystems: deterministic versus stochastic processes. *ISME J* **7**, 1310-1321 (2013).

2. Liu Y, *et al.* Bacterial responses to environmental change on the Tibetan Plateau over the past half century. *Environ Microbiol* **18**, 1930-1941 (2016).

3. Barberán A, *et al.* Continental-scale distributions of dust-associated bacteria and fungi. *Proc Natl Acad Sci U S A* **112**, 5756-5761 (2015).

4. Martiny JBH, *et al.* Microbial biogeography: Putting microorganisms on the map. *Nat Rev Microbiol* **4**, 102-112 (2006).

5. Wetzel RG. *Limnology*. Academic Press (2001).

6. MacArthur RH, Wilson EO. *The theory of island biogeography*. Princeton Univ Pr (1967).

7. Whittaker RJ, Fernández-Palacios JM. *Island biogeography: ecology, evolution, and conservation*. Oxford University Press, USA (2007).

8. Fukami T. Historical Contingency in Community Assembly: Integrating Niches, Species Pools, and Priority Effects. *Annu Rev Ecol Evol Syst* **46**, 1-23 (2015).

9. Hervàs A, Camarero L, Reche I, Casamayor EO. Viability and potential for immigration of airborne bacteria from Africa that reach high mountain lakes in Europe. *Environ Microbiol* **11**, 1612-1623 (2009).

10. Hervas A, Casamayor EO. High similarity between bacterioneuston and airborne bacterial community compositions in a high mountain lake area. *FEMS Microbiol Ecol* **67**, 219-228 (2009).

11. Barberán A, Henley J, Fierer N, Casamayor EO. Structure, inter-annual recurrence, and global-scale connectivity of airborne microbial communities. *Sci Total Environ* **487**, 187-195 (2014).

12. Wang J, Shen J, Zhang L, Liu E. Spatial heterogeneity of oxygen exchange between sediment-water interface in lakes. *J Lake Sci* **21**, 474-482 (2009).

13. Wang J, Shen J, Zhang L, Fan C, Li W, Pan J. Sediment-water nutrient fluxes and the effects of oxygen in Lake Dianchi and Lake Fuxian, Yunnan Province. *J Lake Sci* **22**, 640-648 (2010).

14. Zhou J, Bruns MA, Tiedje JM. DNA recovery from soils of diverse composition. *Appl Environ Microbiol* **62**, 316-322 (1996).

15. Shen J, Zhang L, Guo J, Ray JL, He J. Impact of long-term fertilization practices on the abundance and composition of soil bacterial communities in Northeast China. *Appl Soil Ecol* **46**, 119-124 (2010).

16. Wang J*, et al*. Regional and global elevational patterns of microbial species richness and evenness. *Ecography*, DOI: 10.1111/ecog.02216 (2017).

17. Caporaso JG*, et al*. QIIME allows analysis of high-throughput community sequencing data. *Nat Meth* **7**, 335-336 (2010).

18. Wang J, Soininen J, He J, Shen J. Phylogenetic clustering increases with elevation for microbes. *Environ Microbiol Rep* **4**, 217-226 (2012).

19. Magoč T, Salzberg SL. FLASH: fast length adjustment of short reads to improve genome assemblies. *Bioinformatics* **27**, 2957-2963 (2011).

20. Edgar RC. UPARSE: highly accurate OTU sequences from microbial amplicon reads. *Nat Meth* **10**, 996-998 (2013).

21. DeSantis TZ*, et al*. Greengenes, a chimera-checked 16S rRNA gene database and workbench compatible with ARB. *Appl Environ Microbiol* **72**, 5069-5072 (2006).

22. Caporaso JG, Bittinger K, Bushman FD, DeSantis TZ, Andersen GL, Knight R. PyNAST: a flexible tool for aligning sequences to a template alignment. *Bioinformatics* **26**, 266-267 (2010).

23. Wang Q, Garrity GM, Tiedje JM, Cole JR. Naive Bayesian Classifier for Rapid Assignment of rRNA Sequences into the New Bacterial Taxonomy. *Appl Environ Microbiol* **73**, 5261-5267 (2007).

24. Sanchez G. *PLS path modeling with R*. Trowchez Editions (2013).
